# Subacute PM2.5 Exposure Induces Hepatic Insulin Resistance Through Inflammation and Oxidative Stress

**DOI:** 10.3390/ijms26020812

**Published:** 2025-01-19

**Authors:** Yao Lu, Wenke Qiu, Ruiwei Liao, Wenjuan Cao, Feifei Huang, Xinyuan Wang, Ming Li, Yan Li

**Affiliations:** 1 School of Basic Medical Sciences, Guangzhou University of Chinese Medicine, No. 232, East Waihuan Road, Guangzhou Higher Education Mega Centre, Panyu District, Guangzhou 510006, China; luyao0054@stu.gzucm.edu.cn (Y.L.);; 2 School of Basic Medical Sciences, Guangdong Pharmaceutical University, No. 280, East Waihuan Road, Guangzhou Higher Education Mega Centre, Panyu District, Guangzhou 510006, China

**Keywords:** PM2.5, hepatic insulin resistance, oxidative stress, inflammation, PI3K-AKT signaling pathway, China

## Abstract

Epidemiological studies prove that type II diabetes, characterized by insulin resistance (IR), may be caused by fine particulate matter 2.5 (PM2.5). However, underlying mechanisms whereby PM2.5, particularly during short-term exposure, induces liver dysfunction leading to IR remains poorly understood. In the present study, HepG2 cells and the BALB/c mouse model were used to explore how PM2.5 affects insulin sensitivity. The effects of subacute PM2.5 exposure on glucose metabolism were examined using commercial kits. Oxidative stress and inflammation were detected by fluorescent staining and RT-qPCR. The phosphorylation of PI3K/AKT was examined by Western blot. Subacute PM2.5 exposure induced IR, as reflected by increased glucose levels in cell supernatants, elevated insulin levels, and the impaired intraperitoneal glucose tolerance test in mice. PM2.5 induced oxidative stress, as evidenced by increased reactive oxygen species, cytochrome P450 2E1, and malondialdehyde, along with reduced superoxide dismutase 1/2 and silent information regulator 1. IL-6 and TNF-α were found to be upregulated using RT-qPCR. Western blot showed that PM2.5 inhibited the PI3K-AKT signaling pathway, indicated by the decreased phosphorylation of PI3K/AKT in HepG2 cells. Additionally, H&E staining showed only mild hepatic injury in mice liver. These results firmly suggest that subacute PM2.5 exposure induces insulin resistance through oxidative stress, inflammation, and the inhibition of the PI3K-AKT signaling pathway.

## 1. Introduction

In recent years, the severity of air pollution has received great attention from the Chinese government. Environmental and air quality have been gradually improving due to the implementation of several powerful policies, including reducing pollution emissions and enhancing pollution control. However, short-term severe pollution remains a serious public health concern. For instance, research by Meng et al. has shown that China, especially the seriously polluted North China region, has experienced severe haze events in recent years, which has raised public worries [1]. Their paper indicates that severe outdoor air pollution still persists even though mitigation measures have largely eliminated the most extreme cases. Chen et al. also reported that two specific weather events (dust storms) on March 15 and 28, 2021, led to the acute deterioration of air quality in a short time [2]. So, it is urgent to verify the short-term and severe toxic effects that dust storms have on human health.

PM2.5 refers to atmospheric particulate matter with an aerodynamic diameter ≤ 2.5 μm, primarily originating from the combustion of biomass and chemical fuels [3]. Owing to its small particle size, PM2.5 not only deposits in the distant lung, causing respiratory system damage, but also penetrates the alveoli and enters the bloodstream, leading to multiple systemic disorders, including atherosclerosis, renal injury, and Alzheimer’s disease. For example, Chaulin et al. demonstrated that PM2.5 significantly exacerbates the progression of atherosclerosis and cardiovascular diseases [4]. Zhang et al. confirmed that the intratracheal administration of PM2.5 induced acute kidney injury in BALB/c mice [5]. Wang et al. proved that PM2.5 exposure not only induces age- and Alzheimer’s disease (AD)-related pathological alterations in healthy rodents but also triggers early pathological changes in transgenic AD mice [6]. Research indicates that exposure to PM2.5 is strongly linked to both the development and advancement of multiple diseases.

Diabetes mellitus (DM) has emerged as a major global burden of chronic disease. The International Diabetes Federation suggests that by 2035, the global diabetic population will rise to approximately 500 million [7]. Diabetes primarily manifests in two forms: type 1 and type 2 diabetes, of which type 2 diabetes mellitus is the most prevalent form of diabetes in adults. Insulin resistance, the central pathological feature in type 2 diabetes, is characterized by the reduced responsiveness of insulin-target tissue to insulin stimulation. This condition manifests as decreased insulin receptor numbers and impaired signal transduction, ultimately leading to glucose metabolic disorders. Epidemiological evidence indicates that environmental air pollution represents one of the major risk factors for diabetes [8]. Several studies have demonstrated that long-term exposure to PM2.5 is significantly associated with increased diabetes incidence and prevalence [9,10]. Quantitative analysis indicates that a 10 μg/m^3^ increase in PM2.5 concentration is expected to raise the percentage incidence of diabetes to 15.66% [11]. Recent epidemiological studies further revealed that chronic PM2.5 exposure leads to both an increased risk of conventional diabetes and a higher incidence of early-onset diabetes [12]. These epidemiological findings collectively establish a causal relationship between long-term PM2.5 exposure and diabetes development. In the real world, short-term elevations in PM2.5 concentrations are frequently observed in many regions. However, the current understanding of the impact of subacute PM2.5 exposure on glucose metabolic homeostasis and its underlying molecular mechanisms remains limited, warranting further investigation.

The liver serves as a crucial target organ for insulin and plays a pivotal role in maintaining glucose metabolic homeostasis [13,14]. Hepatocellular injury frequently leads to decreased insulin sensitivity and subsequent insulin resistance. The molecular mechanisms underlying hepatic insulin resistance are intricate and multifaceted, involving multiple stress responses and signaling, such as oxidative stress, inflammation, and the PI3K-Akt pathway. Oxidative stress refers to an imbalance between free radical production and antioxidant defenses. Under oxidative stress, the activity of the antioxidant enzyme system in hepatic tissue, including SOD and SIRT1, is significantly decreased, while the expressions of CYP2E1 and MDA are upregulated, resulting in the disruption of cellular redox homeostasis and the promotion of ROS accumulation. It is widely accepted that oxidative stress in the liver is strongly associated with glucose metabolic disorders [15]. The accumulation of ROS leads to insulin resistance and impaired glycogen synthesis in hepatocytes, ultimately promoting the development and progression of type 2 diabetes [16,17,18]. Moreover, scavenging ROS generation significantly improves hepatic insulin sensitivity, providing compelling evidence for the causal relationship between hepatic oxidative stress and insulin resistance [19].

Meanwhile, hepatic inflammation is also strongly associated with insulin resistance. On the one hand, proinflammatory factors interfere with insulin signaling cascades, thereby attenuating hepatic insulin sensitivity. On the other hand, inflammatory mediators can upregulate the expression of key gluconeogenic enzymes, including PEPCK and G6Pase, thus promoting hepatic gluconeogenesis while inhibiting glucose uptake [20]. Insulin resistance, in turn, can exacerbate the inflammatory state, establishing a self-perpetuating cycle that further accelerates type 2 diabetes progression. Therefore, anti-inflammation is considered an effective therapeutic approach for type 2 diabetes [21].

The PI3K/Akt signaling pathway functions as an essential mediator connecting inflammatory responses, oxidative stress injury, and hepatic insulin resistance. Akt phosphorylation constitutes a pivotal regulatory node for insulin signal transduction, which requires the PI3K-dependent recruitment of phosphatidylinositol-3,4,5- trisphosphate (PIP3). Attenuated Akt phosphorylation disrupts glucose homeostasis and metabolic signaling [22,23,24]. Many studies have found that the proinflammatory mediators IL-6 and TNF-α may directly suppress PI3K activity and subsequently attenuate Akt phosphorylation. This molecular dysregulation ultimately leads to diminished cellular insulin sensitivity [25].

Previous studies have proved that water-soluble ionic compounds carried by PM2.5 can traverse the blood–tissue barrier and accumulate in hepatocytes. Moreover, insoluble particulate matter, mainly composed of heavy metals and polycyclic aromatic hydrocarbons (PAHs), exhibits the capacity to accumulate within and translocate across bronchopulmonary barriers, facilitating systemic distribution and endotoxin release [26]. PM2.5 in the circulation may reach the liver and result in hepatic injury. Based on these and other similar findings, we hypothesize that subacute PM2.5 exposure may induce hepatic oxidative stress and inflammation, which can lead to insulin resistance through the modulation of the hepatic PI3K/Akt signaling pathway.

## 2. Results

### 2.1. Physicochemical Characterization of Particulate Matter

DLS analysis revealed the distinct dispersion characteristics of SRM-1649b in PBS and RPMI-1640 medium (Table 1). In PBS, the mean diameter was 900.5 ± 37.11 nm with a polydispersity index (PDI) of 0.592 ± 0.016, while in RPMI-1640, the diameter was 627.4 ± 12.94 nm with a significantly lower PDI of 0.048 ± 0.021. The zeta potential was measured as −16.4 mV in both media, indicating the moderate colloidal stability of the dispersions. Notably, the extremely low PDI value (0.048) in RPMI-1640 confirmed the excellent monodispersity of the particles in this medium. The morphological characteristics and size distribution of the particles were examined by SEM at 5000× magnification. The SEM results (Figure S1 in Supplementary Materials) showed irregularly shaped particles with sizes predominantly distributed between 349.5 nm and 1.755 μm. Higher magnification imaging (40,000×) clearly showed that individual particles exhibited rough surface morphology with numerous small protrusions. These results, in agreement with other reports, indicated that particle sizes of SRM-1649b were actually below 2.5 μm and could be used as PM2.5.

### 2.2. Cell Viability

**Figure 1:**
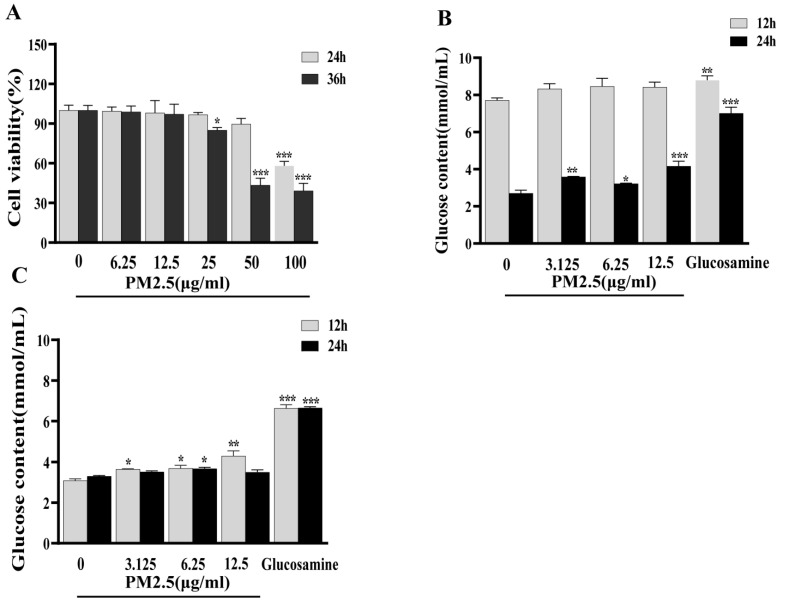
PM2.5 exposure induces insulin resistance in HepG2 cells. (**A**) Cytotoxicity of PM2.5 to HepG2 cells. (**B**) Glucose concentrations in cell culture supernatants of HepG2 cells treated with various concentrations of PM2.5. (**C**) Glucose concentrations in cell culture supernatants after PM2.5 removal and medium replacement. Each experiment was conducted in three replicate wells and repeated independently three times (*n* = 9). Data are presented as mean ± SD. * *p* < 0.05, ** *p* < 0.01, *** *p* < 0.001 versus 0 (control group).

The results are shown in Figure 1A, and it is demonstrated that at concentrations of 0–12.5 μg/mL, cell viability remained above 90% at both 24 h and 36 h exposure times (no significant difference with the control group), indicating no obvious cytotoxicity to liver cells in this concentration range. At 25 μg/mL, a slight decrease in cell viability in HepG2 cells was observed after 36 h of exposure (*p* < 0.05 vs. control group). At higher concentrations (50 and 100 μg/mL), cell viability decreased significantly, particularly after 36 h of exposure (dropping to approximately 40%). At the level of 12.5 μg/mL, PM2.5 does not induce cell death and avoids potential confounding effects from excessive cell death. Therefore, concentrations of 3.125, 6.25, and 12.5 μg/mL were used for subsequent in vitro experiments.

### 2.3. PM2.5 Exposure Induces Insulin Resistance in HepG2 Cells

To investigate the effect of PM2.5 on hepatic insulin sensitivity, HepG2 cells were incubated with PM2.5 for 12 h or 24 h, and then the cell culture supernatants were collected for the glucose concentration measurement. The results showed that after 24 h of PM2.5 stimulation, glucose levels were significantly higher in the PM2.5-treated group than in the negative control group (*p* < 0.05), suggesting PM2.5-induced insulin resistance in HepG2 cells (Figure 1B). To determine whether insulin resistance persisted, the cell culture medium was removed after incubation with PM2.5 for 24 h, followed by three washes with PBS. Then, cells were cultured in fresh medium without PM2.5 for an additional 12 h or 24 h, and the glucose concentration in the supernatants was measured again. The results indicate that glucose levels remained significantly elevated for the PM2.5-treated group at 12 h post-treatment (*p* < 0.05, Figure 1C), confirming the sustained insulin-resistant phenotype in HepG2 cells.

### 2.4. PM2.5 Caused Oxidative Stress in HepG2 Cells

**Figure 2:**
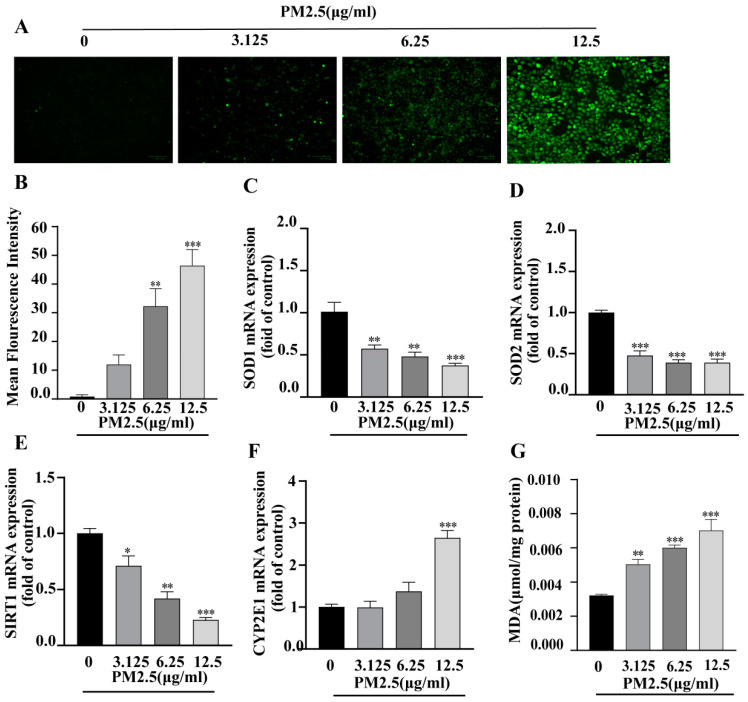
PM2.5-induced oxidative stress in HepG2 cells. (**A**) Representative fluorescence microscopy images showing intracellular ROS production (DCFH-DA fluorescent probe, ×200; scale bar = 100 μm, *n* = 3). (**B**) Quantification of relative fluorescence intensity using Image J (version 1.51j8, NIH, Bethesda, MD, USA). (**C**–**F**) Relative mRNA expression levels of SOD1, SOD2, SIRT1, and CYP2E1 determined by RT-qPCR. (**G**) MDA content determined by colorimetric assay. Each experiment was conducted in three replicate wells and repeated three times independently. Data are presented as mean ± SD (*n* = 9). * *p* < 0.05, ** *p* < 0.01, *** *p* < 0.001 versus 0 (control group).

Oxidative stress is closely associated with the development of insulin resistance; therefore, we evaluated intracellular ROS and oxidative stress markers in PM2.5-stimulated HepG2 cells. Intracellular ROS levels were measured using the DCFH-DA fluorescent probe. The results showed increased fluorescence intensity in PM2.5-exposed HepG2 cells compared to controls (Figure 2A,B), indicating that PM2.5 treatment significantly increased ROS production. Additionally, the expression levels of antioxidant-related genes (SOD1, SOD2, and SIRT1) were analyzed by RT-qPCR. The results demonstrated that these genes were downregulated following PM2.5 exposure, as shown in Figure 2C–E. Moreover, the transcription level of the oxidative stress marker CYP2E1 was significantly upregulated in PM2.5-stimulated HepG2 (Figure 2F). We also noticed that the MDA content of HepG2 increased after PM2.5 exposure (Figure 2G). Collectively, these findings demonstrate that PM2.5 exposure induces oxidative stress in HepG2 cells.

### 2.5. PM2.5-Induced Inflammatory Factor Expression in HepG2 Cells

**Figure 3:**
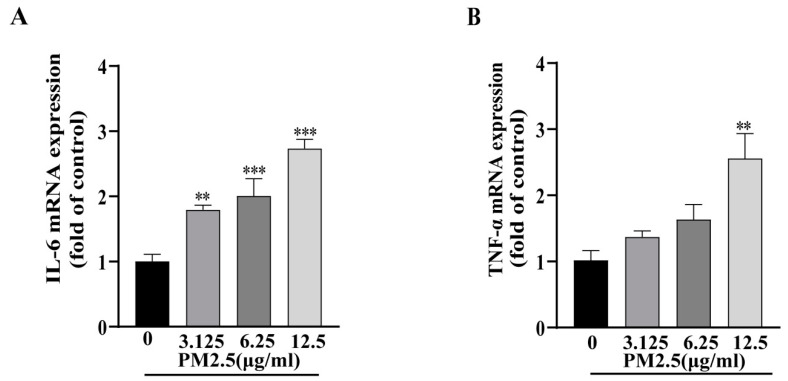
Effects of PM2.5 on inflammatory cytokine expression in HepG2 cells. Relative mRNA expression of IL-6 (**A**) and TNF-α (**B**) determined by RT-qPCR. Data are presented as mean ± SD (*n* = 9) from three independent experiments performed in triplicate. ** *p* < 0.01, *** *p* < 0.001 versus 0 (control group).

Given the crucial role of hepatic inflammation in the development of insulin resistance, we examined the mRNA expression levels of two critical proinflammatory cytokines, IL-6 and TNF-α, using RT-qPCR. Our results revealed the significant upregulation of both TNF-α and IL-6 expressions in 12.5 µg/mL PM2.5-exposed HepG2 cells (Figure 3A,B), indicating that PM2.5 exposure triggers an inflammatory response in hepatic cells.

### 2.6. Effects of PM2.5 Exposure on PI3K/AKT

**Figure 4:**
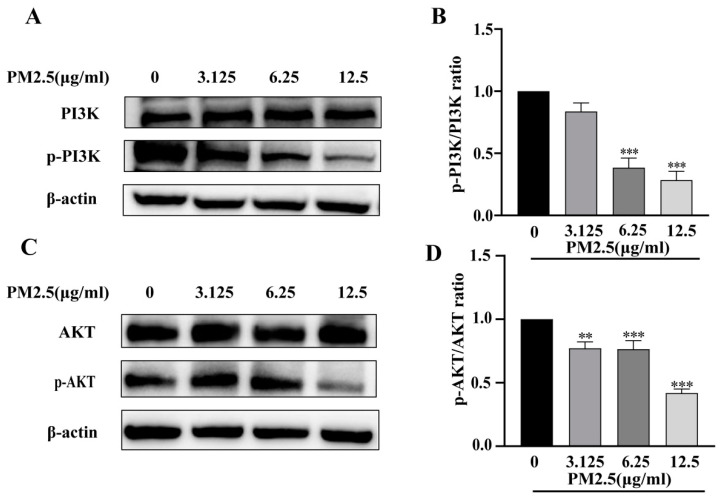
Effects of PM2.5 exposure on PI3K and AKT. (**A**) Western blotting of phosphorylated PI3K and total PI3K. (**B**) Quantification of phosphorylated PI3K/total PI3K ratio. (**C**) Western blotting of phosphorylated AKT and total AKT. (**D**) Quantification of phosphorylated AKT/total AKT ratio. Data are presented as mean ± SD (*n* = 3) ** *p* < 0.01, *** *p* < 0.001 versus 0 (control group).

The PI3K/AKT pathway is considered a well-established indicator of insulin sensitivity. Therefore, Western blot analysis was performed to evaluate the phosphorylation of PI3K and AKT in HepG2 cells following PM2.5 exposure. Our results showed that PM2.5 exposure significantly decreased the phosphorylation levels of PI3K and AKT, as indicated by the reduced ratios of p-PI3K/PI3K and p-AKT/AKT (Figure 4). These findings suggest that PM2.5 may impair insulin sensitivity in HepG2 cells by modulating the PI3K/AKT signaling pathway, thereby leading to cellular glucose metabolism dysfunction.

### 2.7. PM2.5 Exposure Induces Insulin Resistance In Vivo

**Figure 5:**
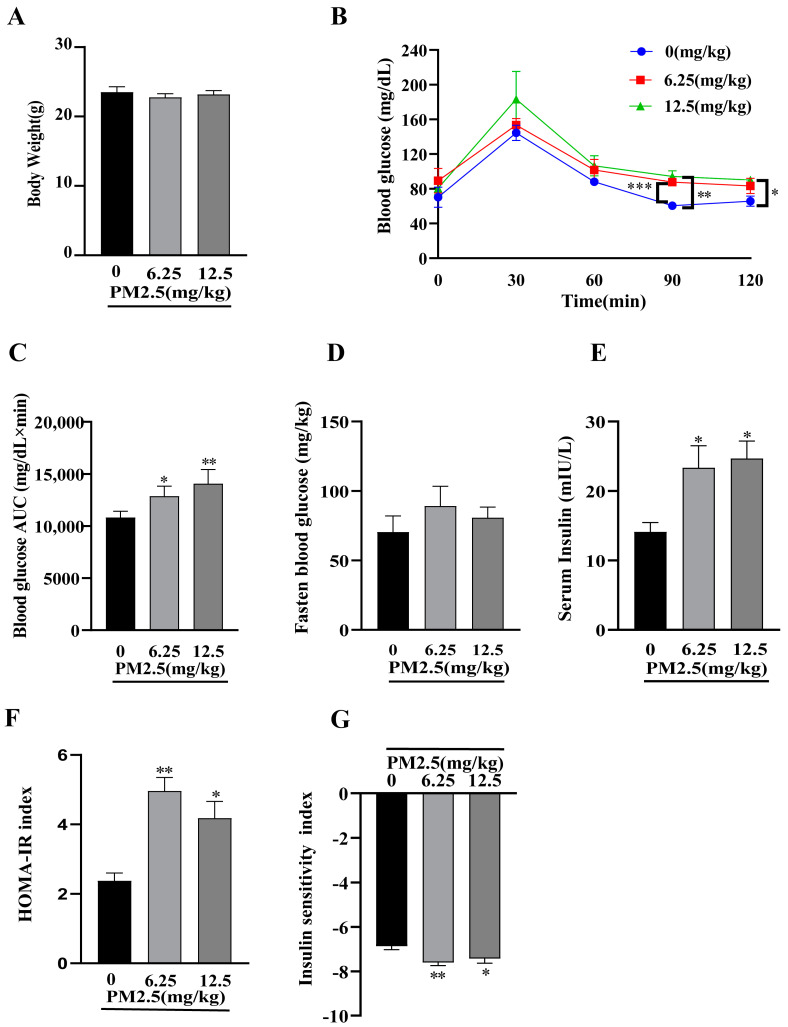
PM2.5 exposure induces insulin resistance in vivo. (**A**) Body weight changes throughout the experimental period. (**B**) IPGTT blood glucose levels and (**C**) corresponding AUC analysis. (**D**) FBG, (**E**) FINS, (**F**) HOMA−IR, and (**G**) ISI values. Data are presented as mean ± SD (*n* = 6). * *p* < 0.05, ** *p* < 0.01, *** *p*< 0.001 versus 0 (control group).

To evaluate the effects of subacute PM2.5 exposure on insulin resistance in vivo, a mouse model was established through PM2.5 injection. Glucose metabolism was assessed by monitoring body weight, Fasting Insulin (FINS), Fasting Blood Glucose (FBG), and an intraperitoneal glucose tolerance test (IPGTT). As shown in Figure 5A, no significant changes in body weight were observed among the groups (*p* > 0.05). IPGTT revealed significantly elevated blood glucose levels in both the 6.25 mg/kg and 12.5 mg/kg PM2.5-treated group at 90 min and 120 min (*p* < 0.01) (Figure 5B). The Area Under the Curve (AUC) analysis, as shown in Figure 5C, demonstrated significantly higher glucose intolerance in the PM2.5-treated group compared with controls (*p* < 0.01). Although the FBG levels (Figure 5D) showed no significant difference from the controls (*p* > 0.05), FINS levels (Figure 5E) were significantly higher in the PM2.5-treated group (*p* < 0.01). The Homeostasis Model Assessment of Insulin Resistance (HOMA-IR) and Insulin Sensitivity Index (ISI) were calculated based on FBG and FINS values. The results demonstrated that PM2.5 exposure significantly increased HOMA-IR while decreasing ISI levels (*p* < 0.01, Figure 5F,G). In aggregate, these results indicate that PM2.5 disrupts glucose metabolic homeostasis in mice, confirming that subacute PM2.5 exposure induces insulin resistance in vivo.

### 2.8. Effects of PM2.5 Exposure on Hepatic Oxidative Stress In Vivo

**Figure 6:**
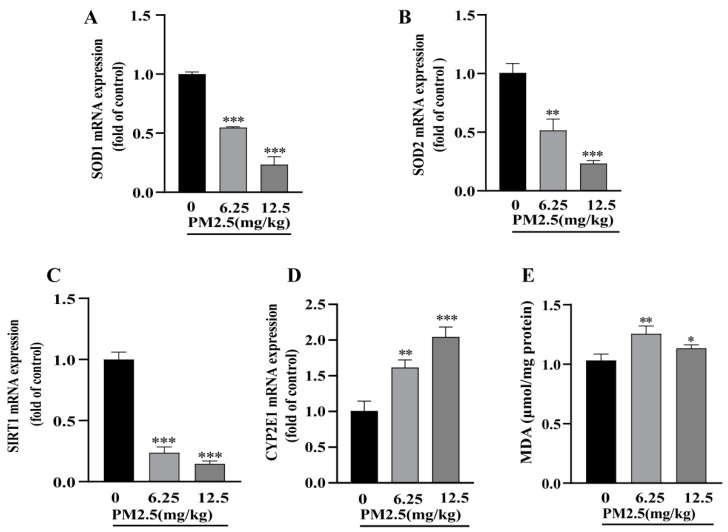
Effects of PM2.5 exposure on hepatic oxidative stress markers in mice. (**A**–**D**) Relative mRNA expression of antioxidant enzymes (SOD1, SOD2, and SIRT1) and CYP2E1 analyzed by RT-qPCR. (**E**) Hepatic MDA content measured by the lipid peroxidation assay. Data are presented as mean ± SD (*n* = 6). * *p* < 0.05, ** *p* < 0.01, *** *p* < 0.001 versus 0 (control group).

Hepatic oxidative stress markers from PM2.5-exposed C57BL/6 liver were then examined by RT-qPCR. As shown in Figure 6A–D, PM2.5 exposure significantly decreased the expression levels of SOD1, SOD2, and SIRT1 while increasing CYP2E1 expression. Additionally, the MDA level was also measured, and the results are shown in Figure 6E. It clearly indicates that PM2.5 exposure considerably promotes hepatic MDA levels. Taken together, these findings demonstrate that PM2.5 induces oxidative stress in the liver.

### 2.9. PM2.5 Exposure Promotes Hepatic Inflammatory Response in Mice

**Figure 7:**
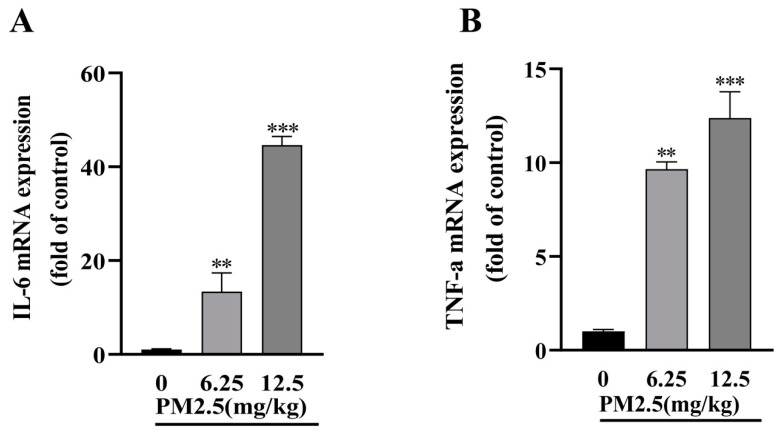
PM2.5 exposure promotes hepatic inflammatory responses in mice. Relative mRNA expression of IL-6 (**A**) and TNF-α (**B**) was determined by RT-qPCR. Data are presented as mean ± SD (*n* = 6). ** *p* < 0.01, *** *p* < 0.001 versus 0 (control group).

We next examined the expression of inflammatory mediators in mouse livers. As shown in Figure 7A,B, PM2.5 exposure significantly increased the mRNA expression levels of IL-6 and TNF-α. These results indicate that subacute PM2.5 exposure induces the production of proinflammatory cytokines in mice liver.

### 2.10. Effects of PM2.5 Exposure on Hepatic Function in Mice

**Figure 8:**
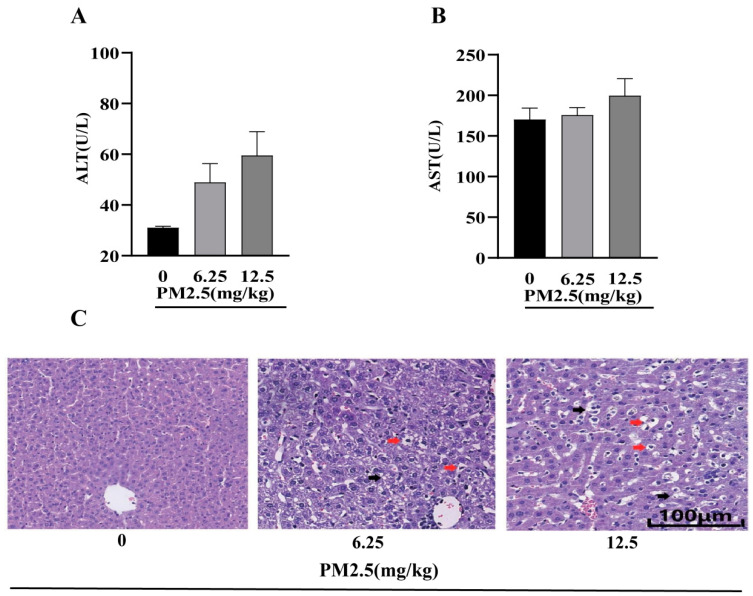
Effects of PM2.5 exposure on liver function and histopathological changes in mice. (**A**) Serum ALT and (**B**) AST levels determined by enzymatic assay. Data are presented as mean ± SD (*n* = 6). (**C**) Representative H&E-stained liver sections (×200; scale bar = 100 μm). Red arrows indicate vacuolar degeneration; black arrows indicate reticular arrangement.

Hepatic injury is often associated with insulin resistance. Therefore, biochemical analysis and H&E staining were performed. As shown in Figure 8A,B, no significant differences in ALT and AST levels were observed among the groups. However, the results of H&E staining (Figure 8C) revealed altered hepatic lobular architecture in PM2.5-exposed mice, characterized by hepatocellular steatosis, cytoplasmic vacuolization, and disrupted reticular fiber arrangement. In conclusion, although PM2.5 exposure did not alter ALT and AST levels, the observed histopathological changes suggested a potential link between PM2.5-induced insulin resistance and hepatic injury.

## 3. Discussion

In this study, we demonstrated that subacute PM2.5 exposure could induce hepatic insulin resistance through in vivo and in vitro experiments. Our findings also revealed that PM2.5 triggered hepatic oxidative stress and inflammatory responses, leading to impaired PI3K/Akt signaling pathway transduction and subsequent insulin resistance.

### 3.1. Significance of Studying Subacute PM2.5 Exposure Effects

Accumulating epidemiological evidence suggests that PM2.5 may serve as a potential risk factor for the initiation and progression of DM [27,28]. It has been reported that approximately 20% of the global type 2 diabetes burden in 2019 can be attributed to PM2.5 exposure [29]. A comprehensive analysis of available clinical observations and laboratory investigations demonstrates that long-term PM2.5 exposure disrupts normal glucose metabolism [30]. Cai et al. and Yang et al. observed that chronic exposure to PM2.5 is associated with increased risks of diabetes and obesity [31,32]. Xu et al. demonstrated that chronic PM2.5 exposure impaired glucose tolerance in C57BL/6 mice [33].

Besides long-term PM2.5 exposure, short-term exposure is closer to real-world scenarios. To cite a case, particulate pollution during the winter heating season, industrial activities, and severe dust storms can lead to rapid increases in the Air Quality Index (AQI) and higher PM2.5 concentrations within short periods [34]. Moreover, in the winter season, as sandstorms in northern China are the main source of PM2.5, it seems more urgent to study the toxic effect of acute or subacute exposure to urban dust. Therefore, the health impacts of short-term high-dose PM2.5 exposure cannot be overlooked. Wei et al. evaluated hospitalization records of 95 million individuals aged ≥65 years and found that short-term PM2.5 exposure was associated with the increased risk of hospitalization for multiple diseases, with this association persisting even on days when PM2.5 concentrations were below the World Health Organization (WHO) ambient air quality guidelines [35]. In a 30-year review, Anderson et al. reported that acute exposure significantly increased the incidence of cardiovascular events [36]. Notably, in a prospective cohort study, Sun et al. enrolled 70 healthy volunteers from Wuhan University and conducted eight follow-up visits using a repeated-measures design [37]. During each visit, individual PM2.5 exposure levels were monitored for three consecutive days, followed by a comprehensive clinical examination on the fourth day. This demonstrated how short-term repeated exposure to PM2.5 over a one-month period led to significant alterations in insulin sensitivity among the participants. However, the effects and mechanisms of subacute PM2.5 exposure on health effects, especially glucose metabolism and insulin homeostasis, remain unclear.

### 3.2. Liver as a Key Target Organ in PM2.5-Induced Insulin Resistance

The liver is a crucial organ mediating the glucose metabolism process. Studies have shown that the liver, a key regulator of insulin sensitivity, is particularly susceptible to PM2.5 exposure. Epidemiological research has demonstrated that ambient PM2.5 exposure is associated with increased incidence and mortality rates of hepatic diseases, including non-alcoholic fatty liver disease (NAFLD) and hepatocellular carcinoma, indicating the detrimental effects of PM2.5 on liver function [38,39]. In this study, we employed both in vitro and in vivo models to investigate the effects of subacute PM2.5 on liver functions and glucose metabolism. Our in vitro studies utilizing HepG2 hepatocytes revealed that PM2.5 exposure markedly perturbed glucose metabolic homeostasis, as evidenced by the aberrant elevation of glucose concentrations in the culture medium. Consistent with these in vitro observations, our in vivo studies demonstrated that PM2.5 exposure elicited comprehensive metabolic perturbations in mice, manifested by significant alterations in key glucose homeostatic parameters, including elevated FINS, abnormal IPGTT, increased AUC, and elevated HOMA-IR, accompanied by a marked decrease in ISI. Moreover, detailed histopathological analyses demonstrated that subacute PM2.5 exposure precipitated substantial architectural modifications in hepatic tissue, primarily characterized by disrupted hepatic cord architecture and compromised tissue integrity. Collectively, these findings firmly indicate that subacute PM2.5 exposure initially triggers hepatic structural and functional abnormalities, subsequently induces liver injury, leading to hepatic insulin resistance, and ultimately results in systemic glucose metabolic imbalance. This proves that impaired liver function is one of the main mechanisms of PM2.5-induced insulin resistance.

### 3.3. Oxidative Stress Mediates PM2.5-Induced Hepatic Dysfunction

Oxidative stress is widely recognized as a fundamental mediator in the pathogenesis of insulin resistance. Chronic PM2.5 exposure activated Nrf2-mediated oxidative stress response and enhanced JNK-dependent inhibitory signaling cascades, ultimately resulting in hepatic insulin resistance [40], which highlights the link between hepatic oxidative stress and insulin resistance.

Studies have demonstrated that PM2.5 exposure induces oxidative stress in various tissues, including the heart, brain, and liver. In the cardiovascular system, for instance, PM2.5 exposure significantly increases levels of oxidative stress markers, such as MDA, leading to endothelial dysfunction [41]. In the brain, studies have shown that following PM2.5 exposure, there is increased ROS production across brain regions, accompanied by decreased antioxidant enzyme activity [6]. Consistent with previous studies, our data demonstrated that subacute PM2.5 exposure induced elevated hepatic oxidative stress, as evidenced by increased ROS and MDA levels in vitro and in vivo.

The molecular mechanisms of PM2.5-induced oxidative stress are multifaceted, differing in different tissues. In hepatocytes, CYP2E1-mediated oxidative stress constitutes a fundamental mechanism underlying the pathogenesis and progression of various liver diseases, thus establishing CYP2E1 as a key molecular determinant in oxidative stress cascades [42]. Additionally, CYP2E1 promotes lipid peroxidation and related processes, thereby exacerbating oxidative stress-induced cellular damage. SOD, as a fundamental antioxidant metalloenzyme, specifically catalyzes the dismutation of superoxide anion radicals (O_2_^•−^) into hydrogen peroxide (H_2_O_2_) and molecular oxygen (O_2_). The upregulation of SOD expression and enhanced catalytic activity can ameliorate oxidative stress conditions. Conversely, decreased SOD expression or inhibited enzymatic activity impairs superoxide radical scavenging, thereby exacerbating oxidative stress. SIRT1 is closely associated with mitochondrial function and attenuates oxidative stress by regulating mitochondrial biogenesis and function. It serves as a regulatory node in the oxidative stress response network by coordinating antioxidant gene expression and ROS detoxification [43]. Notably, reduced SIRT1 expression also leads to intensified oxidative stress. Our research revealed that subacute PM2.5 exposure triggered hepatic oxidative stress by elevating CYP2E1 expression and suppressing the expression of antioxidant factors (including SOD1, SOD2, and SIRT1) in vitro and in vivo. These findings reveal that subacute PM2.5 exposure triggers a cascade of pathophysiological changes through exacerbating hepatic oxidative stress, ultimately resulting in impaired hepatic glucose tolerance. This discovery not only elucidates the potential molecular mechanisms underlying PM2.5-induced pathogenesis but also provides novel insights into therapeutic strategies. Notably, interventions targeting hepatic oxidative stress may represent a promising therapeutic approach to ameliorate hepatic glucose metabolic disorders and consequently alleviate insulin resistance. Establishing the feasibility of this therapeutic strategy warrants further investigation.

### 3.4. Inflammatory Response in PM2.5-Mediated Insulin Resistance

The development of insulin resistance is primarily attributed to proinflammatory cytokine responses in insulin-responsive tissues, particularly the liver and adipose tissue [44]. Proinflammatory factors participate in the pathogenesis of insulin resistance through diverse signaling pathways. Among these, TNF-α could interfere with insulin signaling by inhibiting tyrosine phosphorylation of insulin receptor substrate [45]. Furthermore, Klover, P. J et al. demonstrated that the sustained elevation of IL-6 impairs insulin signaling in hepatic tissues through the activation of suppressors of cytokine signaling 3, leading to insulin resistance [46]. Both clinical and laboratory studies have demonstrated that anti-inflammatory agents or the specific inhibition of proinflammatory factors can enhance hepatic insulin sensitivity [47]. These findings highlight the crucial role of inflammatory factors in the development and progression of type 2 diabetes. The present study observed that acute PM2.5 exposure significantly upregulated the expression of proinflammatory factors IL-6 and TNF-α in HepG2 cells and hepatic tissue. This is consistent with previous studies on chronic PM2.5 exposure, further confirming the crucial role of inflammatory responses in the PM2.5-mediated impairment of hepatic insulin sensitivity. These results suggest that interfering inflammatory responses may represent a potential strategy for reversing PM2.5-associated insulin resistance.

### 3.5. PI3K/AKT Pathway in PM2.5-Mediated Insulin Resistance

The PI3K/AKT signaling cascade serves as a critical mediator in insulin signaling transduction. It orchestrates glucose metabolic homeostasis through the regulation of insulin clearance, glucose transport, and cellular uptake [48]. The activated PI3K/AKT cascade orchestrates the phosphorylation of diverse downstream substrates, thereby sustaining insulin function in insulin-responsive organs. Substantial evidence has established that the dysregulation of the PI3K/AKT signaling axis, particularly the significant attenuation of its phosphorylation level, disrupts glucose transporter 4 translocation, thereby perturbing glucose metabolism and potentially initiating insulin resistance [14]. Notably, accumulating evidence demonstrates that both oxidative stress and inflammatory responses attenuate PI3K/AKT pathway activation, concomitantly suppressing glycogen synthesis while augmenting gluconeogenesis [49]. Our experimental results indicate that subacute PM2.5 exposure markedly diminishes the phosphorylation level of PI3K and its downstream effector AKT, which represents one of the potential mechanisms involved in PM2.5-induced insulin sensitivity decrease.

Finally, we assessed the liver function of PM2.5-exposed mice. Although subacute PM2.5 exposure did not elicit significant alterations in the ALT or AST level, a histological examination revealed subtle histopathological changes in hepatic architecture. These findings contrasted markedly with observations from chronic PM2.5 exposure studies, where prolonged exposure has been shown to induce hepatocyte death [50]. Based on evidence showing the cumulative toxicity of PM2.5 in the liver, we hypothesized that chronic PM2.5 exposure exacerbates severe hepatocellular damage, while subacute exposure primarily alters cellular function, potentially contributing to insulin resistance without inducing hepatocyte death.

### 3.6. Study Limitations and Future Prospects

Several limitations of this study should be addressed. In the development of our mouse model, we administered PM2.5 suspension via tail vein injection rather than using exposure methods that more closely simulate natural conditions. Natural exposure methods, such as whole-body and nose-only inhalation systems, offer significant advantages in simulating real-world PM2.5 exposure scenarios. These systems enable exposure through natural respiratory processes and effectively simulate the complex components and particle size distribution of atmospheric PM2.5. Such methods are particularly suitable for long-term exposure studies, enabling stable exposure over extended periods (days to months), the observation of cumulative effects, investigation of chronic pathological changes, and assessment of adaptive responses.

However, given our focus on early responses and the rapid mechanistic changes following acute PM2.5 exposure, we opted for the tail vein injection method. This approach effectively simulates PM2.5 entry into the bloodstream, enabling a direct toxicity assessment. This method also offers several advantages: precise control over PM2.5 dosage and timing, reduced experimental variability, and enhanced data reproducibility and reliability. It also minimizes the need for repeated anesthesia and surgical interventions. This model has been extensively validated in environmental particulate toxicology studies. For instance, Zhu et al. demonstrated the correlation between PM2.5 exposure and adverse pregnancy outcomes through intravenous administration [51]. Bai et al. confirmed that intravenously administered diesel exhaust particles (DEPs) induced systemic inflammatory responses [52]. A comprehensive review by Shang et al. further substantiated the validity of this administration route in particulate toxicology research [53].

Nevertheless, we acknowledge the limitations of this modeling approach compared to real-world exposure conditions. In future investigations, a model established by intranasal inhalation could be employed to further investigate the biological responses to PM2.5 under natural exposure conditions.

## 4. Materials and Methods

### 4.1. Preparation and Characterization of PM2.5

As sandstorms are one of the primary atmospheric pollution concerns in North China and serve as a main source of PM2.5, urban dust (SRM-1649b), purchased from Sigma-Aldrich (St. Louis, MO, USA) was used in the present study to investigate its toxic effects. It was collected from Washington, D.C., by the National Institute of Standards and Technology (NIST) and has been widely used as atmospheric particulate matter standard reference material in PM2.5-related toxicology studies [54]. SRM-1649b was suspended in phosphate-buffered saline (PBS, pH 7.4) and Dulbecco’s Modified Eagle Medium (RPMI-1640) to prepare stock solutions at 5 mg/mL, which were subsequently diluted to working solutions of 100 µg/mL. Dynamic light scattering (DLS) and a scanning electron microscope (SEM) were used to analyze the particle size characteristics of SRM-1649b. For DLS, samples were sonicated using a probe sonicator (Sonics Vibra™ VC505, Newtown, CT, USA) at 30% amplitude in an intermittent mode (15 s on, 15 s off, and repeated 3 times). Then, hydrodynamic diameter and zeta potential (ζ) were subsequently measured using a Zetasizer Nano ZS system (ZEN3600, Malvern Panalytical Ltd., Malvern, Worcestershire, UK). For SEM, the particles were drop-cast onto silicon wafers and allowed to air-dry at room temperature prior to analysis; then, the morphological characterization of the samples was detected using a scanning electron microscope (SIGMA 360, Carl Zeiss AG, Oberkochen, Germany).

### 4.2. Cell Culture

Human hepatocellular carcinoma HepG2 cells were obtained from Ruishu Biotechnology Co., Ltd. (Guangzhou, China). HepG2 cells were routinely cultured in RPMI-1640 medium (Gibco, Grand Island, NY, USA) supplemented with 10% fetal bovine serum (FBS, Gibco, Grand Island, NY, USA) and antibiotics (100 U/mL penicillin and 100 μg/mL streptomycin) at 37 °C in a humidified atmosphere containing 5% CO_2__._ Glucosamine, an insulin resistance inducer (Cat. No. S1635, Beyotime Biotechnology, Shanghai, China), was dissolved in PBS to a stock concentration of 100 mM and served as a positive control.

### 4.3. Cell Viability Analysis

HepG2 cells were seeded in 96-well plates at a density of 2 × 10^3^ cells per well and incubated overnight. Subsequently, cells were treated with different concentrations of PM2.5 and incubated at 37 °C for an additional 24 h. Cell viability was assessed using the Cell Counting Kit-8 (CCK-8; Fudebio, Hangzhou, China). All experiments were performed according to the manufacturer’s instructions. Cell viability was calculated as follows: viability (%) = [(ODtreated − ODcontrol)/ODcontrol] × 100%, where OD represents optical density at 450 nm.

### 4.4. Establishment of Insulin Resistance Model in HepG2 Cells

HepG2 cells were cultured in 6-well plates for 24 h until they reached 80% confluence. The cells were subsequently exposed to an RPMI-1640 medium containing PM2.5 at specified concentrations (3.125, 6.25, and 12.5 μg/mL). The insulin resistance inducer glucosamine in a 15 mmol/L working concentration was used as a positive control. Following 12 h or 24 h of incubation, culture supernatants were harvested and measured using a commercial glucose detection kit (Nanjing Jiancheng Bioengineering Institute, Nanjing, China) to detect the glucose concentration. All procedures were performed according to the manufacturer’s protocol. The absorbance was measured at 420 nm using a microplate spectrophotometer (Molecular Devices, San Jose, CA, USA).

### 4.5. Detection of Intracellular ROS

HepG2 cells were incubated with various concentrations of PM2.5 for 24 h, followed by incubation with DCFH-DA (Beyotime Institute of Biotechnology, Shanghai, China) at 37 °C for 30 min in the dark. The cells were then washed three times with PBS and observed under a fluorescence microscope (Olympus, Tokyo, Japan). Fluorescence intensity was analyzed using Image-Pro Plus 6.0 software (Media Cybernetics, Bethesda, MD, USA).

### 4.6. Quantitative Real-Time PCR (RT-qPCR) Analysis

The mRNA expression levels of oxidative stress markers (SOD1, SOD2, CYP2E1, and SIRT1) and inflammatory markers (IL-6, TNF-α) in HepG2 cells and mouse liver tissues were analyzed using RT-qPCR. Total RNA was extracted using the TRIzol^®^ reagent (Takara Biomedical Technology, Kusatsu, Japan). The RNA concentration was determined using a NanoDrop 2000 spectrophotometer (Thermo Fisher Scientific, Waltham, MA, USA). First-strand cDNA was synthesized using SuperScript III reverse transcriptase (Invitrogen, Thermo Fisher Scientific, Waltham, MA, USA) following the manufacturer’s instructions. RT-qPCR was performed using the QuantiNova™ SYBR^®^ Green PCR Kit (Takara Biomedical Technology, Kusatsu, Japan). The PCR conditions consisted of initial denaturation at 95 °C for 10 min, followed by 40 cycles of denaturation at 95 °C for 15 s, and annealing/extension at 60 °C for 60 s. The relative gene expression was calculated using the 2^−ΔΔCT^ method. The primer sequences used in this study are listed in Table 2 and Table 3.

### 4.7. Detection of MDA

MDA levels were determined using an MDA assay kit (Beyotime Biotechnology, Shanghai, China). Briefly, cells were washed with PBS after culture medium removal and lysed on ice for 30 min. The supernatant was collected by centrifugation (12,000× *g*, 10 min, 4 °C), and protein concentration was determined using a BCA protein assay kit (Bio-Rad Laboratories, Inc., Hercules, CA, USA). The 0.37% (*w*/*v*) TBA solution, MDA working solution, and standards were prepared according to the manufacturer’s instructions. Samples mixed with agents were heated at 100 °C for 15 min, cooled to room temperature, and centrifuged (10,000× *g*, 5 min). The absorbance of the supernatant was measured at 532 nm using a microplate reader, and MDA levels were calculated based on the absorbance values.

### 4.8. Western Blot Analysis

Cells were lysed in an RIPA buffer (Beyotime Institute of Biotechnology, Shanghai, China) supplemented with protease and phosphatase inhibitors. Protein concentration was determined using the BCA method. The total protein (30 μg) was separated by 10% SDS-PAGE and transferred onto polyvinylidene difluoride (PVDF) membranes. The membranes were blocked with 5% non-fat milk in TBST (TBS containing 0.1% Tween-20) at room temperature for 2 h, followed by overnight incubation with primary antibodies at 4 °C. The primary antibodies included anti-AKT (1:1000; Abcam, UK, Cat. No. ab179463), anti-p-AKT (1:1000; Abcam, UK, Cat. No. ab192623), anti-PI3K (1:1000; Abcam, UK, Cat. No. ab191606), anti-p-PI3K (1:1000; Abcam, UK, Cat. No. ab278545), and anti-β-actin (1:1000; Abcam, UK, Cat. No. ab8226). After incubation, the membranes were washed with TBST and then incubated with HRP-conjugated secondary antibodies (anti-rabbit or anti-mouse IgG) at room temperature for 2 h. Protein bands were visualized using an ECL reagent (Beyotime Institute of Biotechnology) and imaged using a Bio-Rad imaging system (version 2.0; Bio-Rad Laboratories, Inc.). Band densitometry was analyzed using ImageJ software (version 1.8.0; NIH). For relative quantification analysis, all data were normalized to their respective control groups (set as 1) to calculate fold changes.

### 4.9. Animal Experiments

All animal procedures were approved by the Animal Ethics Committee of Guangdong Pharmaceutical University (Approval No.: gdpulacspf 2022022). Eighteen male BALB/c mice were obtained from the Guangdong Medical Laboratory Animal Center. The mice were housed in specific pathogen-free rooms under controlled conditions (temperature: 21 ± 1 °C; humidity: 50 ± 5%) with a 12 h light/dark cycle and free access to filtered water and a standard diet. The dosage of PM25 was calculated, followed by the methods reported in previously published studies [55,56]. As our research focuses on the effect of subacute exposure to PM2.5, the mice were instilled for only 5 days, which led to the need to moderately increase the dosage of PM2.5. In this study, two different levels of PM2.5 were selected based on the correlation between the physiological parameters of mice and the PM2.5 level on severe haze days in Shenyang, one of the biggest cities in North China. It has been reported that the PM2.5 concentration in the severe haze weather of Shenyang reached a maximum value of 1.4 mg/m^3^ in 2019 (Department of Ecology and Environment of Liaoning Province, 2019). Based on adult mouse physiological parameters (body weight: 25g, tidal volume: 0.2mL, respiratory rate: 150 breaths/min, daily air intake: 43L), the total respiratory volume over 5 days is 215L, and the maximum amount of inhaled PM2.5 by a mouse is approximately 0.31 mg. The equivalent single instillation for the high-dose group is estimated to be 12.5 mg/kg based on the average body weight of the mouse. Considering the range of doses used in related studies, one second of the maximum dose, 6.25 mg/kg, was injected as the exposure concentration for the low-dose group.

After one week of acclimation, the mice were randomly divided into three groups (n = 6 per group): the control group, the 6.25 mg/kg PM2.5 exposure group, and the 12.5 mg/kg PM2.5 exposure group. Prior to injection, PM2.5 suspensions were sonicated for 15 s to prevent aggregation. The PM2.5 exposure groups received daily tail vein injections of PM2.5 suspensions for 5 consecutive days, while the control group received an equal volume of saline via the same route. Their body weight was monitored daily throughout the study period. No mortality was observed during the experimental period.

### 4.10. Assessment of Glycemic Parameters

Following 5 days of PM2.5 exposure, mice fasted overnight. FBG levels were measured from the tail vein blood using a glucometer (Sannuo, Hunan, China). FINS levels were determined using ELISA kits (Meimian, Jiangsu, China) according to the manufacturer’s instructions. The HOMA-IR and ISI were calculated using the following formulas: HOMA-IR = FINS × FBG/22.5 and ISI = ln [1/(FINS × FBG)], respectively.

Subsequently, an IPGTT was performed by injecting a 40% (*w*/*v*) glucose solution (2 g/kg body weight) intraperitoneally into all mice. Blood glucose levels were measured at 0, 30, 60, 90, and 120 min post-injection. The AUC was calculated using the following formula: AUC = [(0 min + 30 min) + (30 min + 60 min) + (60 min + 90 min) + (90 min + 120 min)] × 30/2.

Finally, mice were anesthetized with diethyl ether, and blood was collected from the abdominal aorta. The serum was separated by centrifugation at 1000× *g* for 15 min at 4 °C. The supernatant was carefully collected to avoid disturbing the cell layer. After blood collection, mice were euthanized by cervical dislocation, and the liver tissues were excised. Liver tissues were washed with ice-cold saline and divided into two equal portions. One portion was fixed in 4% (*w*/*v*) paraformaldehyde in tissue cassettes for hematoxylin and eosin (H&E) staining, while the other portion was homogenized and centrifuged at 10,000× *g* for 10 min to collect the supernatant for biochemical analysis. Both the serum and supernatant were stored at −80 °C and were protected from repeated freeze–thaw cycles.

### 4.11. Biochemical Parameters Analysis

The levels of ALT and AST in the serum and MDA in the liver tissue homogenate supernatant were determined using commercial assay kits (Nanjing Jiancheng Bioengineering Institute, Nanjing, China).

### 4.12. Histological Observation of Liver Tissue

Liver samples were fixed overnight in 4% paraformaldehyde, embedded in paraffin, and sectioned into 4 μm thick slices. The sections were then stained with H&E following standard protocols. For each sample, fields were randomly selected and photographed under a light microscope.

### 4.13. Statistical Analyses

Statistical analyses were performed using GraphPad Prism software (version 10.1.2). All data were expressed as the mean ± standard deviation (SD). For comparisons among three or more groups, a one-way analysis of variance (ANOVA) followed by Tukey’s post hoc test was performed. Statistical significance was defined as *p* < 0.05.

## 5. Conclusions

In summary, our findings demonstrate that subacute PM2.5 exposure elicits hepatocellular oxidative stress, inflammation, and aberrant PI3K/AKT signaling pathway activation, culminating in hepatic insulin resistance. These findings advance our mechanistic understanding of subacute PM2.5 exposure-mediated insulin resistance. These studies also provide novel strategies and approaches for the prevention and treatment of PM2.5-related metabolic disorders. In the future, except for a deeper exploration of the mechanisms, it is urgent to pay more attention to evaluating the preventive effects of antioxidant and anti-inflammatory agents, particularly natural bioactive compounds such as oleanolic acid, resveratrol, and other active molecules from traditional Chinese herbs to develop targeted hepatoprotective strategies and reduce the toxic effect of PM2.5 in human health.

## Figures and Tables

**Table 1:** Size distribution of SRM 1649b in suspension by DLS.

Size Distribution (nm)		Polydispersity Index (PDI)		Zeta Potential (ζ) (mV)	
PBS	RPMI-1640	PBS	RPMI-1640	PBS	RPMI-1640
900.5 ± 37.11	627.4 ± 12.94	0.592 ± 0.016	0.048 ± 0.021	−16.4 ± 0.681	−16.4 ± 0.761

**Table 2:** List of primers used for HepG2 cells.

Primer	Sequence
SOD1	Forward:5′ATGGCGACGAAGGCCGTGTGCGTGC-3′ Reverse:5′TTATTGGGCGATCCCAATTACACCACAAGCC-3′
SOD2	Forward:5′CTCCCCGACCTGCCCTACGAC-3′ Reverse:5′GCAGGTAGTAAGCGTG-3′
SIRT1	Forward:5′CAATTCCAGCCATCTCTCTGTCAC-3′ Reverse:5′CAACCTGTTCCAGCGTGTCTATG-3′
CYP2E1	Forward:5′TTTGCGGGGACAGAGACCAC-3′ Reverse:5′TCCTTGATGGCAGGGATTCGG-3′
IL-6	Forward:5′GCCTTCGGTCCAGTTGCCTTC-3′ Reverse:5′GTTCTGAAGAGGTGAGTGGCTGTC-3′
TNF-α	Forward:5′CAATGGCGTGGAGCTGAGAGATAAC-3′ Reverse:5′TCTGGTAGGAGACGGCGATGC-3′
β-actin	Forward:5′ GGACTTCGAGCAAGAGATGG-3′ Reverse:5′GGACTTCGAGCAAGAGATGG-3′

**Table 3:** List of primers used for mouse liver tissue.

Primer	Sequence
SOD1	Foward:5′-AACCAGTTGTGTTGTCAGGAC-3′ Reverse:5′-CCACCATGTTTCTTAGAGTGAGG-3′
SOD2	Foward:5′-CAGACCTGCCTTACGACTATGG-3′ Reverse:5′-CTCGGTGGCGTTGAGATTGTT-3′
SIRT1	Forward:5′-TCTTGTGGTTCAGTAGCACCT-3′ Reverse:5′-TCGCAACTATACCCAGAACATAGACA-3′
CYP2E1	Forward:5′-CTCGGTGGCGTTGAGATTGTT-3′ Reverse:5′-GGACCTTTCCCAATTCCTTTCTT-3′
IL-6	Forward:5′CTTCTTGGGACTGATGCTGGTGAC-3′ Reverse:5′TCTGTTGGGAGTGGTATCCTCTGTG-3′
TNF-α	Forward: 5′CGCTCTTCTGTCTACTGAACTTCGG-3′ Reverse:5′GTGGTTTGTGAGTGTGAGGGTCTG-3′
β-actin	Forward:5′-CGTTGACATCCGTAAAGACCTC-3′ Reverse:5′-TAGGAGCCAGGGCAGTAATCT -3′

## Data Availability

The original contributions presented in this study are included in the article/Supplementary Materials; further inquiries can be directed to the corresponding author.

## Supplementary Materials

The following supporting information can be downloaded at https://www.mdpi.com/article/10.3390/ijms26020812/s1.
