# Supplementary material for: Subacute PM2.5 Exposure Induces Hepatic Insulin Resistance Through Inflammation and Oxidative Stress
Source: Int J Mol Sci. 2025 Jan 19;26(2):812. doi: 10.3390/ijms26020812 (PMC11766349; doi:10.3390/ijms26020812)
Supplement: Supplementary file 1 [file ijms-26-00812-s001.zip › ijms-3394477-supplementary.pdf]

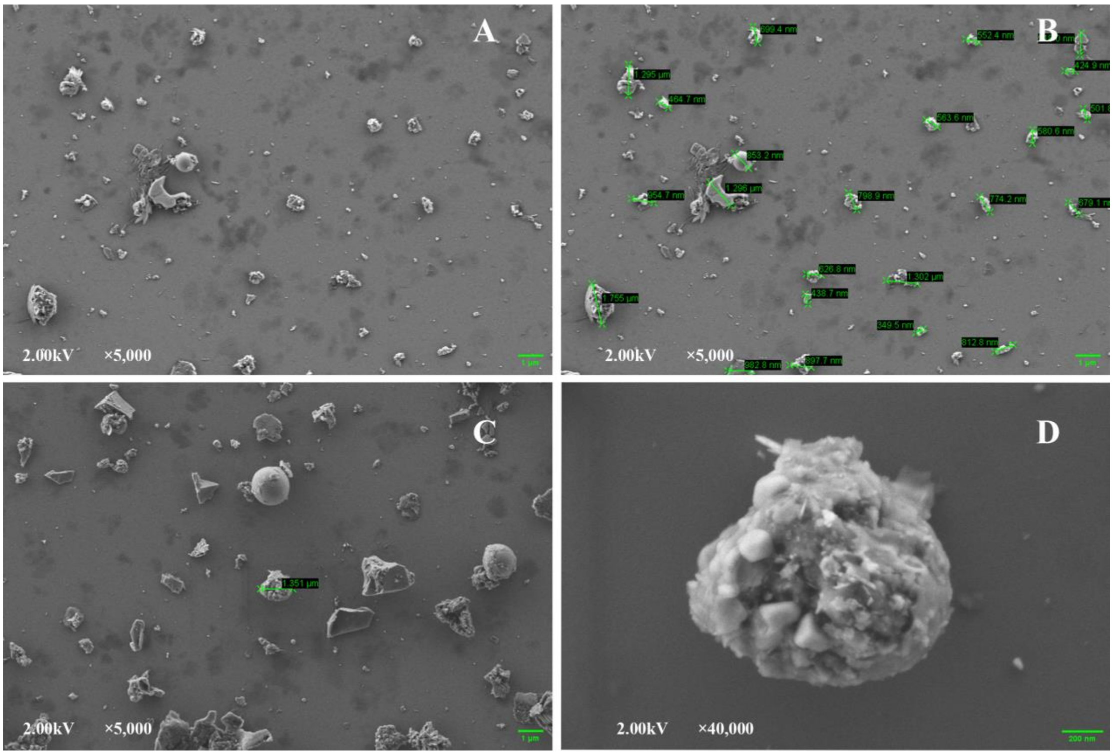

Supplementary Figure S1 Scanning electron microscopy (SEM) characterization of SRM-1649b particles. (A) Representative SEM image showing the overall distribution of particles at 5,000× magnification. (B) Size measurement of individual particles, with dimensions labeled in green (5,000× magnification). (C) Morphological details of particle aggregates (5,000× magnification). (D) High-resolution image of the marked particle in (C) showing detailed surface features (40,000 × magnification). All images were acquired at an accelerating voltage of 2.00 kV.
